# The Pathology of T Cells in Systemic Lupus Erythematosus

**DOI:** 10.1155/2014/419029

**Published:** 2014-04-23

**Authors:** Anselm Mak, Nien Yee Kow

**Affiliations:** ^1^Division of Rheumatology, Department of Medicine, University Medicine Cluster, 1E Kent Ridge Road, Level 10, NUHS Tower Block, Singapore 119228; ^2^Department of Medicine, Yong Loo Lin School of Medicine, National University of Singapore, Singapore 119228

## Abstract

Systemic lupus erythematosus (SLE) is characterized by the production of a wide array of autoantibodies. Thus, the condition was traditionally classified as a “B-cell disease”. Compelling evidence has however shown that without the assistance of the helper T lymphocytes, it is indeed difficult for the “helpless” B cells to become functional enough to trigger SLE-related inflammation. T cells have been recognized to be crucial in the pathogenicity of SLE through their capabilities to communicate with and offer enormous help to B cells for driving autoantibody production. Recently, a number of phenotypic and functional alterations which increase the propensity to trigger lupus-related inflammation have been identified in lupus T cells. Here, potential mechanisms involving alterations in T-cell receptor expressions, postreceptor downstream signalling, epigenetics, and oxidative stress which favour activation of lupus T cells will be discussed. Additionally, how regulatory CD4+, CD8+, and *γδ* T cells tune down lupus-related inflammation will be highlighted. Lastly, while currently available outcomes of clinical trials evaluating therapeutic agents which manipulate the T cells such as calcineurin inhibitors indicate that they are at least as efficacious and safe as conventional immunosuppressants in treating lupus glomerulonephritis, larger clinical trials are undoubtedly required to validate these as-yet favourable findings.

## 1. Introduction

Systemic lupus erythematosus is characterized by the production of plethora of autoantibodies which potentially drive immune-complex related inflammation in various tissues and organs [1]. Breakdown of immune tolerance is believed to be one of the major mechanisms which triggers the production of autoantibodies by B cells and antibody forming cells, leading to inflammation upon binding to autoantigens and consequent tissue damage [2]. As such, SLE was classically thought to be a B-cell driven disease. Recent compelling evidence has demonstrated that T cells are actually crucial in the pathogenesis of SLE in that they enhance the production of autoantibodies by offering substantial help to B cells through stimulating the latter to differentiate, proliferate, and mature, in addition to their support on class-switching of autoantibodies which B cells are expressing [3]. Therefore, SLE is currently believed to be a T cell-driven condition and, indeed, targeting molecules expressed on T cells and their signalling pathways can be one of the potential therapeutic strategies in SLE.

In comparison with healthy subjects, a number of studies have demonstrated that T cells isolated from patients with SLE are abnormal, with regard to their phenotypes and functions [4, 5]. Phenotypic and functional alterations in lupus T cells including expansion of the Th17 population, perturbations of the physiology of T-cell receptors (TCRs) and postreceptor downstream signalling, oxidative stress, and epigenetic changes result in exaggeration of TCR response to stimuli and the propensity of lupus T cells to get activated [6]. Additionally, the failure of the regulatory CD4+ and CD8+ T lymphocytes in alleviating the proinflammatory milieu occurring in SLE is contributory to the pathogenicity of the condition [7, 8]. In this brief review, a detailed account of the putative mechanisms by which the normal physiology of T cells are disturbed and why regulatory T cells fail to alleviate proinflammatory response in SLE will be discussed. The current state of clinical trials evaluating therapeutic agents which target molecules expressing on and inside T cells for the treatment of SLE will be updated.

## 2. T Cells, Their Receptors and Signalling in Normal Situations, and SLE 

### 2.1. T-Cell Receptors and CD3: A Brief Discussion of Their Normal Structures and Functions

T cells recognize antigens presented to them by the major histocompatibility complex of antigen-presenting cells via the TCRs expressed on their surface. Stimulation of TCRs upon antigen binding triggers downstream signalling pathways which enables various physiological functions of the T cells. The majority of TCRs (95%) are heterodimers which compose of an *α* and a *β* chain (*αβ* receptors) and are anchored into the plasma membrane by a short cytoplasmic tail [9]. A minor group (15%) of TCRs comprise a *γ* and a *δ* chain (*γδ* receptors) which are expressed in certain populations of thymic T cells and peripheral T cells in the epithelia [10, 11]. TCRs are associated with CD3 which is a series of polypeptides with consistent amino acid sequences and is responsible for signal transduction upon antigen recognition by the TCRs [9, 12]. CD3 consists of four invariant polypeptides, namely, *γ*, *δ*, *ε*, and *ζ*, and the CD3-TCR complex is arranged in such a way that the four TCR chains (two *α* and two *β* positively charged chains) are associated with two *ε*, two *ζ*, one *γ*, and one *δ* chain polypeptides of the CD3 which are all negatively charged [9, 12]. The CD3 has extracellular, transmembrane, and cytoplasmic tails whereby the 2*ζ* chains (or its variant—the *η* chain) are the longest cytoplasmic chains amongst the rest. The cytoplasmic portions of *ζ* and *η* chains are critically involved in TCR signal transduction for they possess the immunoreceptor tyrosine-based activation motifs (ITAMs) which are targets of phosphorylation by various specific protein kinases in the signal transduction processes [13]. Briefly, ITAMs become phosphorylated in a few minutes following TCR engagement. ITAMs and the subsequent pathways activated, such as the *ζ*-associated protein 70 (ZAP-70) pathway, are essential for T-cell activation [14].

Closely related to CD3*ζ*, Fc*γ*R also associates with the ITAMs. However, instead of stimulating the ZAP-70 pathway, the spleen tyrosine kinase (Syk) pathway is preferentially utilized [15, 16]. Syk stimulation characteristically results in higher calcium influx into cells than that involves the ZAP-70 pathway, probably regulated by transcription factors c-Jun and Ets2 [17]. Such “rewiring” of postreceptor downstream signalling mechanism has a strong pathological implication in lupus T lymphocytes (discussed in the next section).

### 2.2. Alterations in T-Cell Receptors and Their Signalling Pathways

**Figure 1:**
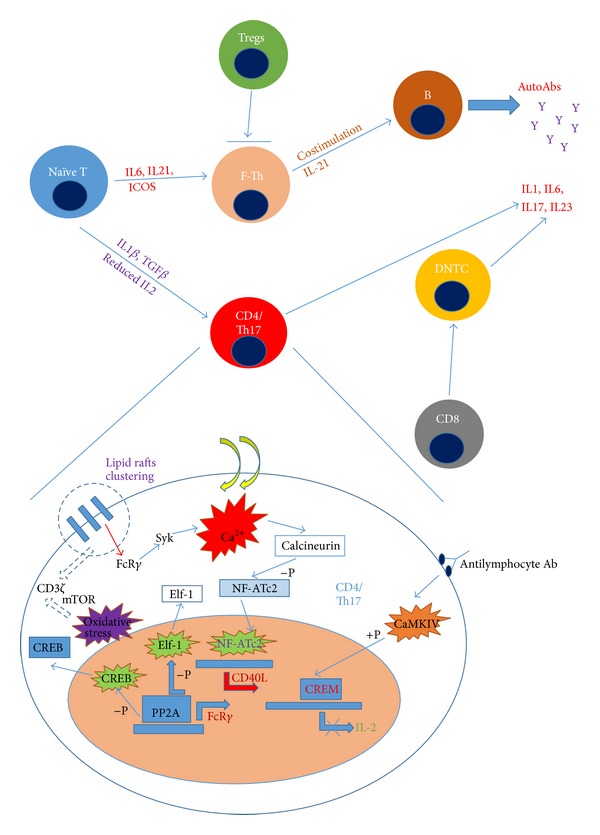
Development of lupus T cells, their interactions with T-regulatory cells and B cells, and alterations of the intracellular physiology of effector lupus T cells. Naïve T cells develop into follicular T-helper cells which cross-talk with B cells for autoantibody production under the stimulation of IL6, IL-21, and ICOS. Naïve T cells develop into effector CD4+ and Th17+ T cells which produce proinflammatory cytokines and exhibit altered intracellular physiology including clustering of CD3-TCR, oxidative-stress induced calcium flux, and consequent change in mRNA transcriptions of various important genes (see text for details). Abbreviations: Tregs, regulatory T cells; ICOS, inducible T-cell costimulator; F-Th, follicular T-helper cells; Syk, spleen tyrosine kinase; CaMKIV, calcium/calmodulin-dependent kinase IV; CREB/CREM, cAMP response element (CRE) binding protein (CREB)/CRE-modulator (CREM); NF-ATc2, nuclear factor of activated T cells; Elf-1, transcription factor Elf-1; Ca^2+^, calcium ion; PP2A, protein phosphatase 2A; mTOR, mitochondrial transmembrane potential and mammalian target of rapamycin; +P, phosphorylation; −P, dephosphorylation.

CD3*ζ* subunits are suboptimally synthetized in T cells from patients with SLE [18]. Moreover, reduction of stability and increase in degradation of CD3*ζ* in lupus T cells are evident [19–21]. To replace the deficient CD3*ζ* subunits, FcR*γ* receptors are reciprocally activated and expressed on lupus T cells [16]. Instead of coupling with ZAP-70 for signalling by the CD3*ζ* subunits, FcR*γ* associates with the Syk pathway and such “rewired” downstream signalling confers stronger phosphorylation of signalling molecules and higher calcium influx which intensifies the TCR-derived signals in lupus T cells [17]. Increase in intracellular calcium activates calcineurin in the cytoplasm which enhances the action of the nuclear factor of activated T cells (NF-ATc2) through the dephosphorylating action of calcineurin [22]. (See Figure 1). Activated NF-ATc2 alters the expression of certain genes including the* CD40L* gene of lupus T cells by binding to the promoter of the* CD40L* gene [22]. CD40L is a costimulatory molecule expressed on T cells and its cognate interaction with CD40 expressed on B cells promotes differentiation, proliferation, and antibody production of the latter, as well as class switching, in conjunction with the action of IL-10 and IL-21 [23].

Another mechanism whereby lupus T cells exhibit a lower threshold of activation is the presence of preaggregated lipid rafts on their cell membrane [24]. The lipid rafts are lipid-rich areas on the cell surface where TCRs and the associated signalling molecules are concentrated [25, 26]. During inactivated state, lipid rafts are evenly distributed throughout the cell membrane but, in lupus T cells, clustering of lipid rafts has been demonstrated even when they are minimally stimulated [24, 27]. Clustered lipid rafts enhance lower threshold of signal transduction as molecules necessary for receptor activations are physically clustered in lupus T cells. To prove these potential mechanisms, intraperitoneal administration of pharmacologically active compounds which disrupt (M*β*CD) and enhance (cholera toxin B) lipid raft clustering demonstrated reduction and promotion of T-cell activation, respectively, in a murine lupus-prone model [28].

### 2.3. Other Abnormal Signalling Pathways in Lupus T Cells

Abnormalities in certain signalling pathways in lupus T cells which lead to defects in T-cell activation in patients with SLE have been increasingly identified. Impairment of cyclic adenosine monophosphate (cAMP-)dependent phosphorylation due to the reduction of protein kinase A levels has been reported [29, 30]. In addition, the activities of pathways involving protein kinase C and p56-lymphocyte-specific protein tyrosine kinase (p56lck) have also been found to be compromised [31, 32]. On the other hand, the activities of protein kinase PKR and phosphatidylinositol-3 kinase (PI3K) were found to be increased in a lupus-prone animal model [33, 34]. Pharmacological inhibition of PI3K can ameliorate glomerulonephritis and decrease mortality in the MRL/Fas^lpr^ murine lupus model [35]. The activity of the mitogen-activated protein (MAP) kinases, which is crucial in the proliferation and apoptosis of T cells, is reduced in T cells of patients with SLE [36]. Animals which are deficit in PKC (an activator of MAP kinase) have been shown to develop spontaneous lupus-like disease [37].

### 2.4. Alterations in Gene Expression Partly due to Reduced DNA Methylation

As described above, upregulation of CD40L in lupus T cells is evident as a result of the activation of NF-ATc2 secondary to high calcium flux [17, 22]. Increase in CD40L upregulates expressions of CD80 and other costimulatory molecules on the antigen-presenting cells which further intensify the stimulatory signals to the T cells [23]. Being a SLE susceptible gene, the* CD40L* gene is methylation sensitive. DNA methylation generally suppresses gene transcription and expression. In SLE, DNA methylation which has been shown to be reduced in T cells, is linked to T cell auto-reactivity [23]. Hypomethylation in one of the X-chromosomes which is inactive in female lupus patients induces overexpression of CD40L mRNA and hence CD40L expression on lupus T cells [23]. Altered MAP kinase and PKC*δ* activities are also caused by hypomethylation secondary to the deficiency of DNA methyltransferase 1 in lupus T cells [23, 38].

IL-2 is essential in reducing the polarization of naïve CD4+ cells towards the Th17 phenotype [39] (see Figure 1). Reduced production of IL-2 demonstrated in patients with SLE enhances the expansion of Th17 population which promotes local inflammation and recruitment of immunocytes in part due to the increased production of IL-17 [40]. Expression of IL-2 by T cells is in fact tightly regulated by the transcription factors cAMP response element (CRE) binding protein (CREB) and the CRE-modulator (CREM) [41]. CREB enhances the transcription of the* IL-2* gene while CREM suppresses it by competing for the CRE binding site with CREB [42]. The balance between CREB and CREM activity, which is important in determining whether IL-2 is upregulated or downregulated, is altered in lupus T cells [43]. The high CREM and CREB ratio in lupus T cells contributes to IL-2 deficiency [43]. There are at least 2 proposed mechanisms to explain the increased CREM and reduced CREB activities in lupus T cells. First, high levels of antilymphocytic antibodies in patients with SLE activate calcium/calmodulin-dependent kinase IV (CaMKIV) which enhances CREM activity through phosphorylation [44]. Second, the increased intranuclear level of protein phosphatase 2A (PP2A) in lupus T lymphocytes dephosphorylates and inactivates CREB [45, 46]. One point of note is that Elf-1, an important transcription factor of CD3*ζ*, is dephosphorylated by the increased level of intranuclear PP2A in lupus T cells [47]. Dephosphorylated Elf-1 fails to associate with the DNA and initiate transcription of CD3*ζ* transcription, leading to the increased FcR*γ* and CD3*ζ* ratio [47], favouring subsequent activation of the Syk instead of ZAP-70 pathways in lupus T cells [17] (see Figure 1).

Increase in oxidative stress has been demonstrated in lupus lymphocytes as evidenced by ultrastructural changes in the form of tubuloreticular structures of organelles in lymphocytes from patients with active lupus [48]. Oxidative stress induces nitric oxide activity and elevation of mitochondrial transmembrane potential which lead to activation of the protein kinase named mitochondrial transmembrane potential and mammalian target of rapamycin (mTOR) in lupus T cells [49]. Increase in mTOR activity causes RAB4A-mediated CD3*ζ* downregulation and results in high calcium flux when lupus T cells are stimulated [50]. Increase in intracellular calcium activates cAMP response element modulator (CREM) which inhibits IL-2 and enhances IL-17 expressions. These changes favour T_h_1 to T_h_17 polarization and inhibit CD8+ T cells [51]. mTOR activation also suppresses FoxP3 transcription by inhibiting DNA methyltransferase 1 (DNMT1) which results in hypomethylation of the FoxP3 promoter [51]. Rapamycin, an inhibitor of mTOR, was demonstrated in a small clinical study of nine lupus patients to be able to normalize T-cell activation-induced calcium influx and reduce overall lupus disease activity [50]. Other potential mechanisms of mTOR in immune response inhibition will be discussed in a subsequent section.

## 3. Alteration in the Number and Suppressor Activity of Regulatory T Cells in SLE

### 3.1. CD4+ T Regulatory Cells

CD4+ regulatory T cells (CD4+ Tregs) were shown to be reduced in the secondary lymphoid organs of the NZB/W F1 lupus-prone mouse model as compared with age-matched nonautoimmune mice [52]. Deficiency of CD4+ Tregs is linked to the development of lupus-like disease, while adoptive transfer of CD4+ Tregs slowed the progression of renal disease and reduced mortality in NZB/W F1 mice [52]. Besides thymic CD4+ Tregs, peripheral-induced CD4+ Tregs (CD4+ iTregs) conferred by the action of IL-2 and TGF*β* were shown to be able to reduce serum anti-dsDNA levels and alleviate immune complex glomerulonephritis secondary to the reduction of T-cell help to B cells in NZB/W F1 mice [53].

In humans, the number of CD4+ Tregs was generally found to be lower in patients with active SLE as compared with those with inactive disease and healthy individuals [54]. Reduced levels of forkhead box P3 (FoxP3) in CD4+ Tregs in patients with active lupus are generally believed to be the reason why these patients have less Tregs-suppressive activity than their counterparts with inactive disease [55–57]. Interestingly, effective immunosuppressive therapies with glucocorticoids and rituximab have been shown to restore the number of functional Tregs in patients with SLE [58–60]. Despite the prevailing belief of the inferior quantity and functional quality of Tregs in patients with SLE, the lack of truly reliable markers which allow identification and isolation of the genuine Treg population renders reliability and reproducibility of Treg studies in SLE an issue [61]. Helios, which is a transcription factor that belongs to the Ikaros family, has recently been shown to be expressed by most of the FoxP3+ T-cells in humans and it has been demonstrated to be able to upregulate FoxP3 expression by binding to the FoxP3 promoter [62]. In contrast to the previous findings which advocated the lower quantity of Tregs in lupus patients with more active disease, the population of Foxp3+ Helios+ Tregs was indeed shown to be significantly expanded in patients with active SLE when compared with those with inactive disease and healthy controls [61, 62]. In addition, the FoxP3+ Helios+ T cells isolated from 20 lupus patients were shown to have lower IL-2 and IFN*γ* productions when compared with those from FoxP3+ Helios− T cells [62].

### 3.2. CD8+ T Regulatory Cells

In both the NZB/W F1 and human monoclonal anti-DNA-induced experimental mouse models, expansion of CD8+ Tregs by tolerogenic peptide suppressed anti-dsDNA production, CD4+ T cell proliferation, and type-2 interferon production, probably as a result of TGF*β* and FoxP3 produced by the CD8+ iTregs [63, 64].

Similar to the findings of CD4+ Tregs, studies addressing the number of circulating CD8+ Tregs in patients with SLE have yielded inconsistent results [65, 66]. CD8+ Tregs from patients with active SLE failed to suppress effector T cells, while CD8+ Tregs from patients with inactive SLE demonstrated comparable suppressive ability as those from healthy individuals [65]. Of particular note, since the data of CD8+ Tregs in SLE are based on a small number of clinical studies, more robust studies are required to further characterize the quantity and functional aspects of CD8+ Tregs in patients with SLE.

### 3.3. *γδ* T Regulatory Cells

Recently, a group of rare *γδ* T cells which express high levels of CD25 and CD27 and low level of CD45RA has been found to possess regulatory and suppressive activities (CD27^+^CD45RA^−^ *γδ* Treg cells), particularly the V*δ*1 subset [67]. Enumeration of the peripheral blood mononuclear cell (PBMC) populations revealed a significantly lower number of circulating CD27^+^CD45RA^−^ *γδ* Treg cells in patients with SLE as compared to that of healthy controls [67]. Furthermore, a significant inverse correlation was found to exist between lupus disease activity and the level of circulating CD27^+^CD45RA^−^ *γδ* Treg cells [67].* In vitro* experiment confirmed the ability of lupus CD27^+^CD45RA^−^ *γδ* Treg cells to express FoxP3 in a CD27-dependent fashion when the cells were cultured in the presence of TGF*β* [67]. In addition, CD27^+^CD45RA^−^ *γδ* Treg cells were demonstrated to be able to suppress the proliferation of autologous effector CD4+ cells in coculture systems [67]. Though rare in the PBMC population, further experiments are required to fully characterize the phenotype and function of these *γδ* Treg cells which may play an important immunopathogenic, as well as potential therapeutic, roles in suppressing the disease activity of SLE.

## 4. Therapeutic Trials Testing Drugs Which Manipulate T Cells in SLE

### 4.1. Calcineurin Inhibitors

The most commonly used calcineurin inhibitors including cyclosporin and tacrolimus have been proven in randomized controlled trials to be at least as efficacious and safe as conventional treatment for proliferative and membranous lupus glomerulonephritis [68–70]. A one-year quasirandomized trial revealed proteinuria remission rates of 83%, 60%, and 27% in patients who were in the cyclosporine, intravenous cyclophosphamide, and prednisolone groups, respectively, although the relapse rate of proteinuria was higher in patients receiving cyclosporine than those who received cyclophosphamide [68]. As an induction therapy, the combination of prednisolone and intravenous cyclophosphamide (a total of six 4-week pulses starting at 750/m^2^ of body surface area) or tacrolimus (starting at 0.05 mg/kg/day and being titrated to a trough level of 5–10 ng/mL) has been shown to be equally efficacious in achieving complete renal remission [70]. Tacrolimus appeared to be safer as adverse events including leucopenia and gastrointestinal complaints were less frequent as compared to subjects in the cyclophosphamide group [70].

### 4.2. Anti-CD40L

As discussed previously, CD40L, which is overexpressed on lupus T cells, stimulates CD40 expressed on B cells to produce autoantibodies. Antagonization of CD40L is thus a potential therapeutic target for the treatment of SLE. Two main clinical trials testing the blockade of the CD40-CD40L pathway in the treatment of SLE are, however, disappointing [71, 72]. In addition to the failure of satisfying the predefined study end-points, the unfavourable side-effect profile of anti-CD40L unfortunately led to the premature termination of a multicentre phase II trial of BG9588 in SLE [72]. In a double-blind, placebo-controlled trial, 85 patients with mild to moderately active SLE were randomized to receive 6 infusions of anti-CD40L at doses of 2.5, 5, and 10 mg/kg and placebo at 0, 2, 4, 8, 12, and 16 weeks [71]. After 20 weeks of treatment, lupus disease activity improved in all groups from baseline but no statistical significance was detected amongst the different groups [71]. No difference in fatigue score and quality of life was noted either [71]. In the smaller phase II, open-label trial evaluating BG9588 in the treatment of 28 patients with proliferative lupus glomerulonephritis, the occurrence of 2 myocardial infarctions in the subjects led to premature termination of the trial although significant reduction of proteinuria, haematuria, and anti-dsDNA titre with increase in serum C3 levels were demonstrated [72].

### 4.3. Rapamycin

Being a safe and well-tolerated drug clinically used for preventing transplant rejection, rapamycin, a macrolide antibiotic which regulates mitochondrial transmembrane potential and calcium influx, was evaluated in a small uncontrolled trial for its effectiveness in patients with SLE [50]. In 9 lupus patients who were refractory to conventional treatment, rapamycin 2 mg daily reduced the disease activity and prednisolone requirement [50]. Mitochondrial calcium level and T-cell activation-induced calcium fluxing were normalized in rapamycin-treated patient [50]. In a recent prospective open-label study, rapamycin was shown to inhibit IL-4 production by and necrosis of double negative (DN) T cells in patients with SLE. In addition, rapamycin enhanced FoxP3 expression in CD25+/CD4+ T-cells and expansion of CD25+CD19+ B cells [73], signifying that mTOR can trigger IL-4 production by and necrosis of DN T cells in active SLE.

### 4.4. N-Acetylcysteine

Recently, N-acetylcysteine (NAC), the precursor of glutathione, was shown in a small clinical trial that at doses 2.4 gm and 4.8 gm daily it could reduce lupus disease activity and fatigue after 3 months of treatment as compared with placebo [74]. NAC reduced mTOR activity and enhanced apoptosis of T cells, accompanied by reversed expansion of the CD4/CD8 populations. Interestingly, NAC was shown to induce FoxP3 expression in CD4+ Treg cells and reduce serum anti-dsDNA levels [74]. Larger clinical trials are certainly required to validate the efficacy of this exciting therapeutic agent, especially it is anticipated that adverse effects of NAC due to immunosuppression are very minimal.

## 5. Conclusion

In both murine system and human disease of SLE, T cells are found to be abnormal based on their alterations in the phenotype, receptor and signalling physiology, gene transcription, and perturbed suppressor activities of regulatory lymphocytes. The substantial involvement of T cells in the pathogenesis of SLE and the apparent success in therapeutics directing at T cells in patients with SLE lead to the firm belief that SLE is indeed a T-cell driven autoimmune disease. While manipulating the B cells and their families with the use of B-cell depleting therapy (BDT) appears very promising in the treatment of SLE and it is argued that B cells are relatively more important in the pathogenesis of SLE than other immunocytes, the discrepantly prolonged beneficial effects of BDT against the much shorter half-life of rituximab invariably explain the potential importance of the participation of T cells in the pathogenic process of SLE [58, 59].
